# Foliar Fungal Endophyte Communities of Scottish Plantation Pines

**DOI:** 10.3390/jof11020148

**Published:** 2025-02-14

**Authors:** Amanda L. Jones, Joanne E. Taylor, Richard A. Ennos

**Affiliations:** 1 Royal Botanic Garden Edinburgh, 20A Inverleith Row, Edinburgh EH3 5LR, UK; 2 Institute of Evolutionary Biology, University of Edinburgh, Ashworth Building, King’s Buildings, Edinburgh EH9 3JT, UK; rennos@ed.ac.uk

**Keywords:** *Anthostomella*, Corsican pine, lodgepole pine, *Pinus contorta*, *Pinus nigra*, *Pinus sylvestris*, Scots pine, tree disease, Xylariales

## Abstract

The diversity of foliar fungal endophyte communities was examined in three economically and ecologically important pine species in Scotland: Scots pine, Corsican pine and lodgepole pine. Two plantation sites comprising all three species were selected in climatically contrasting parts of Scotland and were sampled in late spring by collecting healthy needles from two age classes. Surface sterilisation was carried out before obtaining cultures of fungal isolates, and representatives of common sterile morphotypes were sequenced to determine taxonomic placement. Overall relative proportions of the dominant taxa across sites, tree species and needle age classes were as follows: *Anthostomella* spp. (52%), *Lophodermium seditiosum* (17%) and *Desmazierella acicola* (7%). Many other less frequent taxa were recovered. The results agreed with previous endophyte studies in that the combined effects of site and tree species produced unique endophytic fungal assemblages. Phylogenetic analyses revealed potential sub-species variation associated with site in *Anthostomella pinea*. Our findings point to the potential naturalisation of European fungal endophytic species (e.g., *Anthostomella* spp.) in Scottish pine plantations, particularly in association with Corsican pine.

## 1. Introduction

Research into endophytic fungi has increased significantly in the last 40 years, with conifer studies being extensive compared to those of many other plant lineages [1,2]. These trees present considerable diversity of endophytic fungi, primarily due to their evergreen nature and the corresponding accumulation of endophytes over time. This is in contrast with the lower biodiversity that would be expected, given their typically high latitudes and boreal locations [3]. Conifer endophyte research has focused on natural populations (e.g., [4,5,6,7,8]), in addition to plantation situations (e.g., [9,10,11,12,13,14,15,16,17]). Observations often accompanying these studies include the distribution of endophytic fungi between hosts and localities [17,18,19,20,21,22,23,24,25,26], across needle ages [10,27,28], and seasonal and temporal variations in populations [18,28,29]. Endophytic fungal communities of *Pinus* have been particularly well-studied throughout the northern hemisphere where indigenous pine populations occur (e.g., [4,5,11,13,17,20,21,30,31,32,33]) including in the UK [7,16,34].

The present study aimed to investigate the diversity of foliar fungal endophytes in three economically and ecologically important pine species in Scotland: Scots pine (*Pinus sylvestris*), Corsican pine (*Pinus nigra* ssp. *laricio*) and lodgepole pine (*Pinus contorta* var. *latifolia*). Scots pine is the world’s most widely distributed conifer [35] and is native to Scotland and Europe. It belongs to the genus subsection *Pinus*. Scots pine forms the iconic Caledonian forests and is planted as a valuable timber tree, and it has been grown in large plantations in Scotland since the late 18th century [36]. Corsican pine is native to Corsica and like Scots pine, it belongs to subsection *Pinus* [36]. It was introduced to the UK in 1759 and over the last 60 years has become a well-established forestry species [37]. It is particularly susceptible to disease in the UK and for this reason, a moratorium on planting the species was issued in 2006 [38,39]. It is affected by *Heterobasidium* butt rot, *Lophodermella* needle disease, and shoot dieback caused by *Brunchorstia* [40,41] and it is the primary host for *Dothistroma* needle blight (DNB) in England [42]. Lodgepole pine belongs to the genus subsection *Contortae*. It is native to western North America, where it has a vast natural range and shows similarly large variation in form and vigor depending on provenance [43]. The species was introduced to the UK in 1853 and has become a widely established timber species in the last 60 years [37]. Lodgepole pine is susceptible to an increasing number of diseases and is known as a primary host of DNB in Scotland [42].

Very little research has been carried out on endophytic fungal communities of Scots pine in the UK, and almost nothing is known about Corsican pine and lodgepole pine in this regard. However, the increasing incidence and severity of DNB in these pine species [38], including in Scots pine in Caledonian pine forests [44], has prompted efforts to find out more about their associated foliar fungi.

This study sets out to document the endophyte communities of three pine species (one native and two non-native) co-occurring at two different sites. Analyses of resulting data will be used to elucidate differences in abundance, taxonomic diversity, and composition of endophyte communities amongst host tree species, between sites, and between needle ages. Novel taxa not previously found at native Scots pine sites in the UK will be identified and their phylogenetic relationships with related taxa established.

## 2. Materials and Methods

### 2.1. Sites and Sampling

Two sites of mixed planted pine trees were sampled, each containing the three host species. Torrs Warren Wood, 202.6 ha. of pine plantation, was sampled on 11 May 2016 and is located in southwest Scotland near Stranraer, in Dumfries and Galloway (54.863650, −4.8810159). Tentsmuir Forest (approximately 170 miles northeast), representing 1569 ha. of commercial pine plantation, was sampled on 12 May 2016 and is located approximately 10 km southeast of Dundee on the east coast of Scotland (56.426368, −2.869404) (Supplementary Materials Figure S1). Both sites are managed by Forestry and Land Scotland (FLS) (formerly Forestry Commission Scotland) and have similarly aged trees with comparable areas of natural regeneration. Environmental data were not collected during this study, but a comparison of weather data shows that an average of 1100 mm of rain falls each year in the vicinity of Torrs Warren (with higher monthly averages from October to January), and the mean minimum temperature is 6 °C (1991–2020 data for the West Freugh weather station [45]). InTentsmuir, the average annual rainfall is considerably lower at 713 mm (with little contrast in the monthly averages throughout the year) but the mean minimum temperature is similar (5.3 °C) (1991–2020 data for the Leuchars weather station [45]).

A transect of approximately 400 m was sampled at each site (Supplementary Materials Table S1, Figures S2–S7). Ten trees from each of the three tree species were sampled per site, with two healthy-looking needles selected from each of two needle age classes (years one and two) (10 trees × 3 species × 2 sites × 2 age classes × 2 needles = 240 needles). As Scots pine in Torrs Warren only presented first-year needles, 20 fewer needles were collected, giving a total of 220. Tree age was not recorded, but several studies have suggested that seedlings host a different endophyte community compared to young and adult trees [7,46], so only trees that could be sampled at breast height were collected and they represented a mixture of young and adult trees. Needle age class was determined by identifying terminal bud scars produced in previous years (as in [18]). Some needle litter was also collected to sample saprobic fungi and common endophytic fungi in their saprobic stage. All samples were refrigerated at 4 °C until processing, which took place within 48 h of collection.

### 2.2. Surface Sterilisation and Culturing

The needle samples (220) were surface sterilised and incubated to obtain endophytically occurring fungi. Surface sterilisation took place as follows: 1 min in 70% EtOH, 5 min in 3.5% sodium hypochlorite solution, and then 30 s in 70% EtOH. Five needles were lost during processing, leaving a total of 215 needles for culturing. After sterilisation, each needle was cut into five segments measuring ca. 5 mm and arranged on Petri dishes of malt extract agar (MEA; Fluka, Sigma Aldrich, Darmstadt, Germany) supplemented with 0.3 g/L streptomycin sulphate (Sigma Aldrich, Darmstadt, Germany), which were incubated for two weeks at room temperature.

Isolated cultures were morphotyped based on characteristics following the method of Lacap et al. [47]. Only a few of the fungal isolates sporulated during the study period. These isolates were observed under a light microscope, enabling some species identifications. Numerous fungi were recorded as ‘indeterminate’ as these morphotypes appeared infrequently or were lacking determinate features (i.e., they became overgrown by other fungi). Representative examples of morphotypes were subbed onto fresh MEA plates to obtain pure cultures for DNA extraction. They were left to grow in the same conditions as the original isolates.

Samples of needle litter collected during the initial field study were incubated on damp tissue in covered Petri dishes at room temperature and examined after one week. Microscopic investigations were carried out on the developing fungi, and single-spore isolations were undertaken for a specimen of *Anthostomella* and the resulting strains were sequenced.

### 2.3. DNA Extraction, PCR Amplification and Sequencing

Thirty-four morphotypes were identified. Thirty-two isolates were then sequenced, representing 11 of the most common morphotypes (Supplementary Materials Table S2). DNA extraction was carried out using a Qiagen DNeasy Plant Mini Kit (Qiagen, Hilden, Germany) following the manufacturer’s instructions with the following modifications. In the first step, the initial buffer solution was added to the sample with the addition of a small amount of sterilised sand before grinding with an electric drill using sterilised Eppendorf pestles. The samples were then placed for 1 h on a Thermomixer C (Eppendorf, Hamburg, Germany) at 800 rpm and 65 °C. Finally, in the elution step, two additions of a reduced volume (75 μL) of elution buffer were added to achieve a higher DNA concentration.

Most of the subbed morphotypes were simply barcoded with ITS rDNA (ITS) using fungal-specific primers ITS1F [48] and ITS4A [49], whereas for the single-spore isolations, a fragment of the gene for β-tubulin (*Tub*2) was also sequenced with primers TUB2Fd and TUB4Rd [50], as was nrLSU (LSU) with primers LROR [51] and LR5 [52]. The PCR reaction mixtures and conditions were as outlined in the study by Anderson-Stewart et al. [53] for ITS and *Tub*2, and the study by Rehner & Samuels [51] for LSU. The remaining primers were removed by adding 2 µL of ExoSAP-IT (Cleveland, OH, USA) to 5 µL of the PCR product. Sequencing reactions were conducted for both primers with 1 µL of clean product using a BigDye Terminator v3.1 Cycle Sequencing Kit (Applied Biosystems, Waltham, MA, USA) following the protocol provided. The PCR products were sequenced at Edinburgh Genomics (http://genomics.ed.ac.uk/). The resulting bidirectional sequences were trimmed, assembled, and edited in Sequencher v. 5.1 (2016). Consensus sequences were then queried against existing sequences in NCBI GenBank and UNITE [54] to estimate taxonomic placement (Supplementary Materials Table S2). Similar sequences of *Anthostomella* species were used to build sequence alignments (Table 1).

### 2.4. Phylogenetic Analyses of Anthostomella Species

Phylogenetic analyses were conducted on morphotypes that matched various *Anthostomella* spp. in GenBank (using the BLAST algorithm [55]) and UNITE [54] to help determine their identity (Table 1). ITS, LSU, and *Tub*2 reference sequences were imported from GenBank, based on the ‘*Anthostomella*’ clade *sensu* Voglmayr et al. [56]. There are no close relatives to *Anthostomella* aff. *pinea* in GenBank that have a formal taxonomic status; a sequence was therefore chosen from a study of endophytic fungi on *Pinus ponderosa* in Arizona, USA (Ariz_B328 [57]), as it clustered with *Anthostomella* aff. *pinea* in a provisional phylogenetic analysis. Due to the quality of the few *Tub*2 sequences that were available in GenBank for *Anthostomella* strains, it was decided to proceed with ITS and LSU only. One *Tub*2 sequence is available for AM1 (AM1.1, PV008782). *Microdochium lycopodinum* (Jaklitsch, Siepe & Voglmayr) Hern.-Restr. & Crous (CBS 122885) and *M. phragmitis* Syd. & P. Syd. (CBS 285.71) were chosen as outgroup taxa [56]. They were combined with consensus sequences of three representative *Anthostomella* endophyte strains (ITS only) plus the one saprophyte strain (ITS and LSU) from the present study. ITS and LSU datasets were aligned using MAFFT v.7 [58] and manually edited in MEGA 11 [59]. The regions were separately analysed using maximum likelihood (ML) in the IQ-TREE web server [60] with default settings. Since they showed congruence, the two regions were combined to form a concatenated data matrix in MEGA 11 [59] and set as independent partitions for analysis. Phylogenetic analysis was performed using ML and Bayesian inference implemented in Geneious Prime v.2023.2.1 [61]. An ML tree was reconstructed with RAxML v. 8 [62], using a GTR-GAMMA substitution model with 1000 bootstrap replicates. Bayesian tree inference was carried out using MrBayes v.3.2.6 [63]. For the Bayesian analysis of the dataset, the GTR model was selected. Two independent runs of 1,100,000 generations were run with sampling every 200th generation, with the first 110,000 samples discarded as burn-in for the combined dataset. Posterior probabilities (PP) were used as Bayesian branch support for the consensus tree. Bayesian PP were added to the ML consensus tree which was generated in FigTree v.1.4.4 [64].

### 2.5. Statistical Analyses of Endophytic Fungal Communities

The number of endophytes isolated from each needle sample was calculated (with one needle represented per plate), giving data for four needle samples per tree (with needle age classes examined as well). Needle position (1–5) was noted, but not considered further. After the identification of endophytes was performed, the same counts were made for the number of taxa. Supplementary Materials Table S3 provides the number of isolates and distribution of taxa across the two sites for each of the three tree species. From these data, the number of isolates per sample and the number of fungal taxa per sample were derived. ANOVA [65] was used to investigate whether these two measures differed between sites, amongst tree species, or between the two needle age classes.

### 2.6. Abundance and Diversity of Endophytes

A General Linear Model (GLM) was performed, with site and tree species and the interaction of the two as fixed factors and with the tree (site + tree species) nested within the site and tree species as a random factor.

The effect of the lack of second-year needles for Scots pine in Torrs Warren was considered by examining mean taxa frequency and total isolates for first-year needles only at both sites for all three tree species. The analyses (one-way ANOVA) showed significant differences only for total isolates for the site (*p* = 0.027) and for the mean number of taxa per tree species (*p* = 0.009), which agrees with the overall results presented below.

Tukey’s multiple comparison test was used in the ANOVA to provide 95% confidence intervals for pairwise comparisons between the mean number of taxa found for tree species at each of the sites. Interaction plots and box and whisker plots were generated to illustrate the effect of site and tree species on endophyte taxa and total isolates.

Shannon Index (H’) [66] was used to calculate endophytic fungal diversity for each tree species. This method was chosen as it is an incidence-based measure that accounts for abundance and the evenness of species found in a sample. Evenness was considered an important measure in this study to reflect the degree to which individuals were distributed amongst taxa. Low H’ values would therefore indicate that one or a few species dominate, while high values would show relatively equitable numbers of individuals for each fungal taxon [67]. ANOVA was carried out using calculations derived from the number of taxa per tree (from four needles). They were made with fungal taxa treated separately for each tree species at each site; tree species data were also combined across sites to provide a picture of the effect of tree species (e.g., native vs. non-native origin) on fungal endophyte diversity.

### 2.7. Comparing Communities of Endophytes

Simple correspondence analyses (CA) for the ordination of categorical data were performed with R Statistical Software v.4.4.1 [68] using the packages *FactoMineR* and *factoextra*. This was carried out to visualise the relationships between communities of endophytes found in each of the eleven unique combinations of: site, tree species and needle age (e.g., TWSP1 representing: first-year needles for Scots pine in Torrs Warren = column values). A chi square of independence was performed to indicate the relationship significance between the rows and the columns [69,70].

Incidence-based similarity of endophytic fungal diversity between the three tree species was measured using the Sørensen–Dice Index [71,72]. This measure was calculated using the number of fungal taxa per tree (based on four needles) in order to compare the fungal communities of two tree species in terms of shared and unique endophytic fungi.

## 3. Results

### 3.1. Taxa Isolated

Endophytic fungi were recovered from all but one of the 215 needle samples (99.5%), with a total of 1288 isolates. The isolates were categorised into 34 morphotypes using the criteria outlined in the Materials and Methods section. To confirm the identification of the most common morphotypes, 32 isolates were sequenced, which represented 11 morphotypes (Supplementary Materials Table S2). Table 2 summarises the endophytic fungi isolated and their abundance according to tree species and site. In terms of diversity, the distribution of taxa amongst needles was irregular, with several exclusive to one site (e.g., *Anthostomella* sp., *Lophodermium pinastri* (Schrad.) Chevall. and *Dothistroma septosporum* (Dorog.) M. Morelet only isolated from Tentsmuir) or less often to a particular tree species (e.g., *Cenangium ferruginosum* Fr. only isolated from Corsican pine). A few taxa habitually produced multiple colonies on single needle segments, significantly increasing abundance (e.g., *Anthostomella pinea* and *A.* aff. *pinea* often produced two to four colonies per needle segment). Lodgepole pine showed the most diversity and the biggest disparity between abundance depending on site (Table 2; Supplementary Materials Table S3). The endophyte diversity and abundance for Scots pine in Torrs Warren was lowest due to the lack of second-year needles sampled. Taxonomic assignment to species was possible for 8 of the 34 original morphotypes; the remainder were assigned to genus/family or are listed as the ‘unidentified’ morphotypes, and isolates that were rare, overgrown, or contaminated were assigned ‘indeterminate’ (Table 2). Four classes were represented: Dothideomycetes, Leotiomycetes, Pezizomycetes and Sordariomycetes. Morphological studies enabled the identification of *Sordaria fimicola* (Roberge ex Desm.) Ces. & De Not. and *Sydowia polyspora* (Bref.) E. Müll., whereas *Dothistroma septosporum*, *Lophodermium pinastri,* ‘*Preussia*-like’, and *Xylaria* sp. were initially determined based on culture characteristics, and the remaining identifications of some of the cultures were based on DNA sequence data (Supplementary Materials Table S2). *Anthostomella* spp. and *Lophodermium seditiosum* Minter et al. constituted 69% of total isolates. The site-specific *Anthostomella pinea* Crous and *A.* aff. *pinea*, accounted for 43% of the isolates cultured. *Anthostomella pinea* was especially common to lodgepole pine in Torrs Warren. The needle pathogen, *Lophodermium seditiosum,* was the second most frequent isolate (17%), with the highest numbers being recovered from Scots pine at each site.

### 3.2. Factors Affecting the Abundance of Isolates per Needle

**Figure 1:**
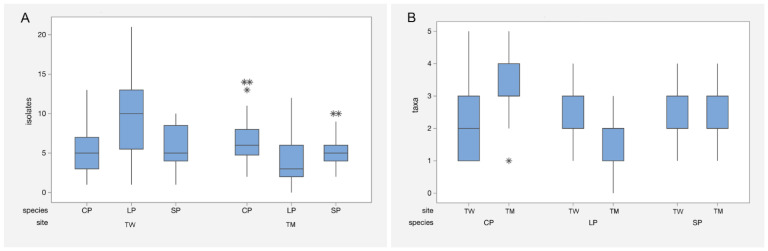
Box and whisker plots of total isolates (**A**) and taxa (**B**) recorded per needle for each tree species at each site. CP = Corsican pine, LP = lodgepole pine, and SP = Scots pine; TW = Torrs Warren and TM = Tentsmuir. The median taxa/total isolate values are shown, and the upper and lower limits of the boxes represent 25–75% of the data, with whiskers providing minimum and maximum values. Outliers are represented by asterisks: * = mild outlier (>1.5 times the interquartile range (IQR)); ** = moderate outlier (>2 times the IQR); *** = extreme outlier (>3 times the IQR).

An ANOVA GLM to evaluate total isolates per needle showed that site had a significant impact on isolate numbers (*p* = 0.006) (Supplementary Materials Table S4). The combination of site and tree species was highly significant (*p* < 0.001), showing a strong interaction between these factors on the number of isolates per needle. Needle age was not found to be a significant factor affecting the number of isolates per needle (*p* = 0.930). Lodgepole pine had the highest mean number of isolates per needle in Torrs Warren (9.0, SE 0.822), reflecting the abundance of *A. pinea* isolations from this tree species at this site, but the lowest mean number of isolates per needle at Tentsmuir (4.026, SE 0.457). Variations between the mean number of isolates for Scots pine and Corsican pine were more moderate amongst the sites (Figure 1A).

### 3.3. Factors Affecting the Number of Endophyte Taxa per Needle

Our ANOVA results showed a strong interaction between site and tree species (*p* = 0.001) for the mean number of endophyte taxa per needle (Supplementary Materials Table S5). This indicates that both of these factors strongly contributed to the diversity of endophyte taxa found. Tree species on its own also significantly affected endophyte taxa (*p* = 0.001). Needle age, however, was not found to have a significant effect on taxa per needle (*p* = 0.895). The mean number of taxa per needle was highest in Corsican pine in Tentsmuir (3.237, SE 0.162) and lowest in Lodgepole pine (1.923, SE 0.124) at the same site. Tukey’s comparison tests (according to 95% confidence interval groupings) showed that the number of taxa per needle in Torrs Warren showed no significant difference in means amongst pine species, while at Tentsmuir, the mean number of taxa per needle differed significantly between all pine species. Box and whisker plots (Figure 1B) illustrate this disparity and show homogeneity in the number of taxa isolated per needle from Scots pine at both sites, perhaps partially due to missing year-two needles, which host greater numbers of endophytic fungi.

An ANOVA GLM to assess the effect of the factors site and tree species on endophyte diversity index (H’) showed that site had a significant effect on the respective H’ values (*p* = 0.032), while tree species did not (*p* = 0.100). The disproportionate abundance of *A. pinea* strongly influenced host tree species diversity values, especially in lodgepole pine, giving it a low diversity value in Torrs Warren (H’= 0.917; Supplementary Materials Table S6). Corsican pine in Tentsmuir showed the highest diversity, indicating a more even distribution and abundance of endophytic taxa (H’ = 2.096).

### 3.4. Needle Age Preference of Endophyte Taxa

**Figure 2:**
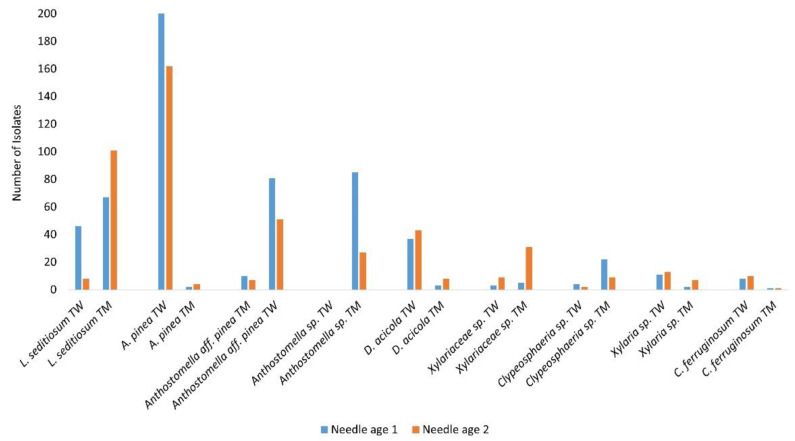
The number of isolates of the main endophytic fungal taxa at each site according to the two needle classes (year one = blue; year two = orange). TW = Torrs Warren; TM = Tentsmuir. *Anthostomella* sp. was not isolated from any tree species at Torrs Warren.

Although needle age affected neither the number of endophyte taxa nor the number of isolates per needle, there was evidence for differences between firstand second-year needles in the occurrence of certain endophyte taxa (Figure 2; Supplementary Materials Table S4). *Lophodermium pinastri* was found only in year-two needles. Xylariaceae sp. (29/31) had higher isolation rates in year-two needles compared to year-one needles (67% and 84% higher for Torrs Warren and Tentsmuir, respectively). The reverse was seen in *Anthostomella* sp., *A. pinea*, and *A.* aff. *pinea* having a much higher abundance in the younger needles.

### 3.5. Differences in Communities of Endophyte Taxa and Site/Tree Species/Needle Age

**Figure 3:**
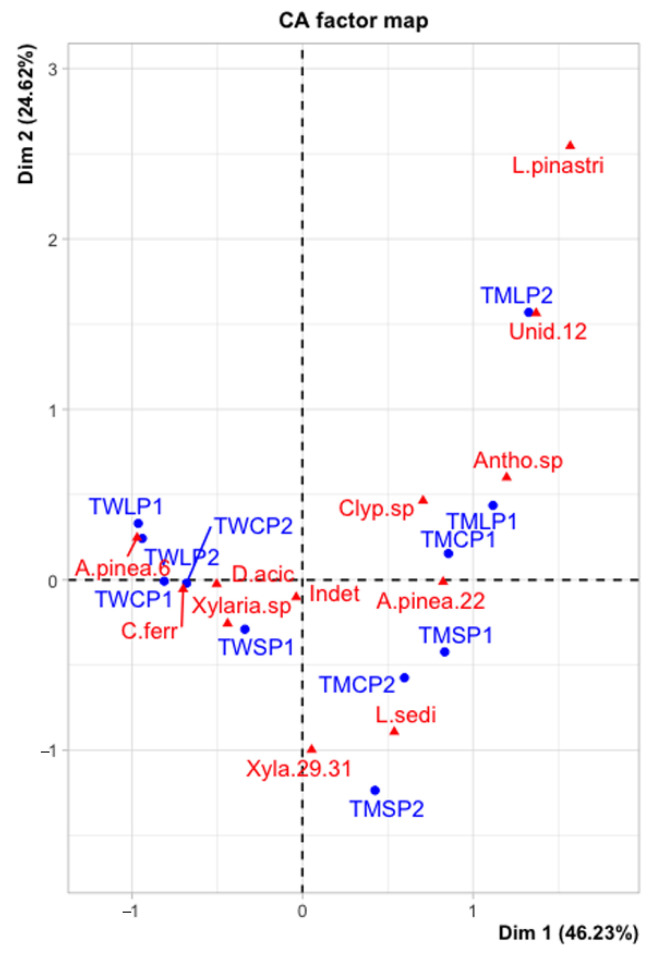
Results of ordination through simple correspondence analysis, with variables comprising the following: site/ tree species/needle age (coded with corresponding letters for site, followed by tree species, and then ‘1’ or ‘2’ to denote needle age); endophyte taxa occurring at relative frequencies greater than 1% (A.pinea.6 = *Anthostomella pinea*; A.pinea.22 = *A.* aff. *pinea*) (Table 2). Total inertia explained by the first two axes is 70.85% (combined eigenvalues of 46.23% and 24.62% for dimensions 1 and 2, respectively). Sites are shown by red triangles/font and species by blue circles/font. TM = Tentsmuir and TW = Torrs Warren; SP = Scots pine, CP = Corsican pine, and LP = lodgepole pine.

The results for the ordination through simple correspondence analysis are shown in Figure 3. The chi square of independence between taxa abundance and site/tree species/needle age combination was equal to 1781.42 (*p* < 0.001; DF = 110), indicating that there is a significant and non-random relationship between the two sets of variables. The contributions of variables to each of the two axes appear in Supplementary Materials Figure S8. The first axis clearly separates the two sites, whereas the second axis separates lodgepole pine and Scots pine, with Corsican pine data points spread over the axis and somewhat closer to Scots pine. The scatter of species is more pronounced in the Tentsmuir data points. Inter-distance between the two sets of variables should not be strictly interpreted; thus, significant groupings corresponding to needle age and taxa abundance were broadly observed in the following associations (using only data points contributing significantly to dimensions): TMLP1 with *Anthostomella* sp.; TMLP2 with *L. pinastri* and Unidentified sp. 12; TMSP2 with Xylariaceae sp. (29/31), *L. seditiosum*; and TWLP1 with *A. pinea*; TMCP1 with *Clypeosphaeria* sp.

The Sørensen–Dice Index of Similarity produced a narrow range of values, suggesting high overall similarity of endophytic fungal communities (Supplementary Materials Table S7). As an index value of 1.0 indicates complete similarity, the Corsican pine–lodgepole pine comparison at Tentsmuir showed the least similarity with 0.688. However, the same combination of species shared the most similarity to Torrs Warren with an index of 0.833. Values for tree species across combined sites showed even more species similarity, with Scots pine–Corsican pine being the most similar in terms of fungal endophyte diversity (0.914).

### 3.6. Phylogenetic Analyses

**Figure 4:**
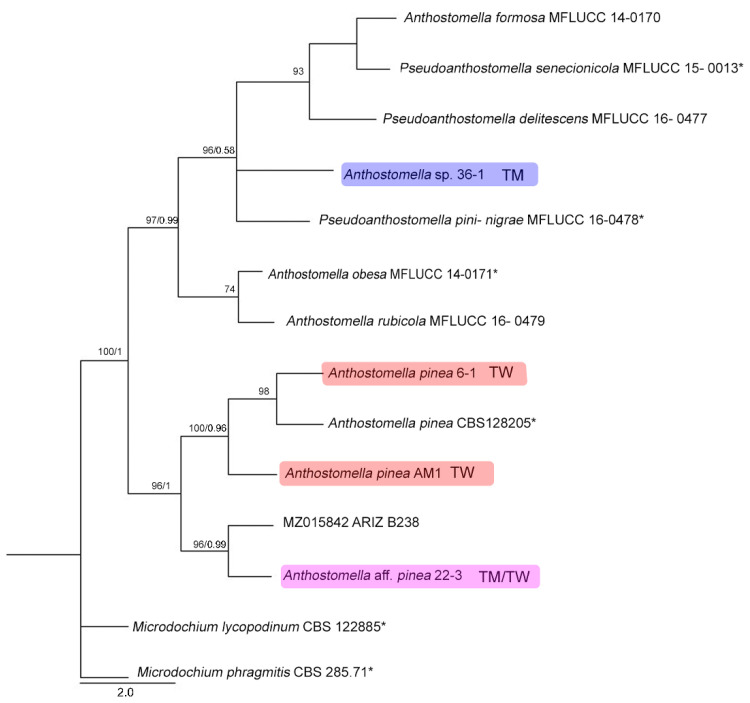
Maximum likelihood (ML) tree based on the concatenated dataset of ITS and LSU sequences of ‘*Anthostomella*’ clade *sensu* Voglmayr et al. [56]. ML bootstrap values (>70) and Bayesian posterior probabilities (>0.90) are given above supported branches. *Microdochium lycopodinum* and *M. phragmitis* were chosen as the outgroup. TM = Tentsmuir; TW = Torrs Warren. Species occurrence is colour coded: Tentsmuir = blue; Torrs Warren = red; both sites = purple; * = type specimens. See Table 1 for GenBank Accession numbers for the sequences used in this phylogeny; see Supplementary Materials Table S2 for the ITS sequences of additional strains of *Anthostomella* spp. recorded in the present study but not included in this phylogeny.

**Figure 5:**
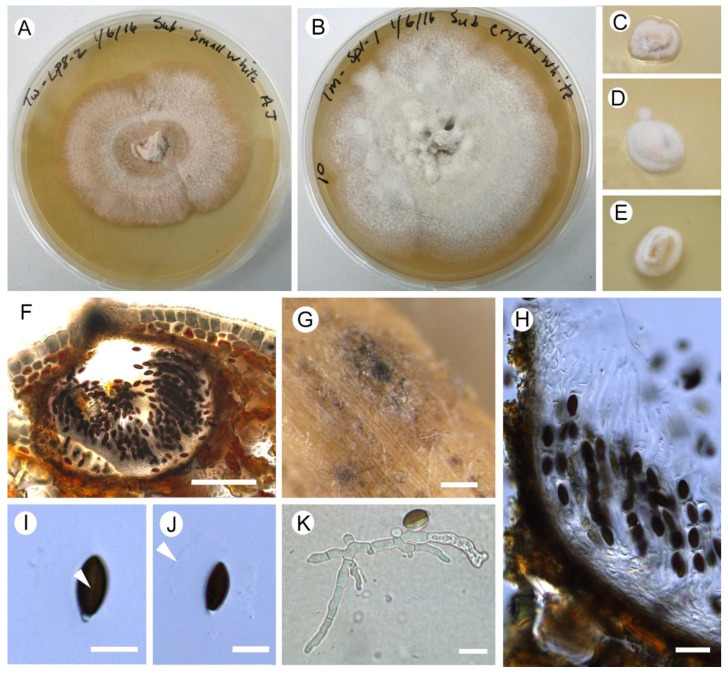
*Anthostomella* spp. (all cultures incubated on a bench top at room temperature). (**A**) *Anthostomella pinea* TWLP8-2 strain 6 (60 mm after 78 d). (**B**) *A.* aff. *pinea* TMSP1-1 strain 22-3 (85 mm after 78 d). (**C**) *A. pinea* TWLP4a-1 strain 6-1 (13 mm after 14 d). (**D**) *A.* aff. *pinea* TWSP1a-1 strain 22-4 (16 mm after 9 d). (**E**) *Anthostomella* sp. TMCP3a-2 strain 36-3 (15 mm after 9 d). (**F**–**K**) *Anthostomella pinea* AM1, E01278543 (Corsican pine litter needles, Torrs Warren forest, 12 May 2016, Amanda Jones). (**F**) Section of sub-epidermal ascoma showing reduced clypeus at the ostiole and lack of stroma. (**G**) Ascoma immersed in the host needle. (**H**) Peridium comprising two layers and showing paraphyses above asci in a gel layer. (**I**) Ascospore showing germ slit (arrowed). (**J**) Ascospore with a mucilaginous sheath (arrowed). (**K**) Germinating ascospore showing opening at the germ slit and end cell retained. Scales: (**F**,**G**) = 100 µm; (**H**) = 20 µm; (**I**–**K**) = 10 µm.

The analysis (Figure 4) produced several well-supported clades. Sequences of the *Anthostomella pinea* morphotypes from this study generally appear to be site-specific including the strains from Torrs Warren obtained through single-spore isolation (sample AM1, held at the Royal Botanic Garden Edinburgh (E01278543)) (Table 2, Figure 5). The two *Anthostomella pinea* morphotypes also differ in their culture characteristics (Figure 5), and appear to be separate species. The remainder of the *Anthostomella* clade *sensu* Voglmayr et al. [56], including *Anthostomella* sp. from the present study (Figure 5), shows a lack of resolution.

## 4. Discussion

In this study, the endophytic fungal communities of three species of pine (Corsican pine, lodgepole pine and Scots pine) planted at two different sites in climatically contrasting parts of Scotland were analysed. Relative proportions of the dominant taxa isolated from healthy, green, surface-sterilised needles across sites, tree species and needle age classes were *Anthostomella* spp. (52%), *Lophodermium seditiosum* (17%) and *Desmazierella acicola* (7%). A complex picture emerged of the combined effect of site and host tree species on the endophytic fungal communities recorded. This was especially evident with *Anthostomella pinea*, which was dominant at Torrs Warren, particularly associated with lodgepole pine, compared to *Anthostomella* sp., which occurred only at Tentsmuir. A third species, *Anthostomella* aff. *pinea* occurred across both sites (but in greater frequencies at Tentsmuir) and all tree species. *Lophodermium seditiosum* was predominantly associated with Scots pine at both sites and *L. pinastri* was virtually absent at both sites. The important pine pathogen *Dothistroma septosporum* accounted for < 0.2% of the total isolates recorded (occurring on only lodgepole pine at Tentsmuir). The distribution of endophytes across needle ages in this study showed definite patterns for certain endophyte taxa, although needle age did not have a significant influence on endophyte communities.

### 4.1. Endophytic Fungal Communities

The endophytic fungal communities recovered in the present study demonstrate an abundance of one or two dominant species with many less common taxa, which is a common observation across endophyte studies [73]. The community composition was influenced by a combination of site and host tree species specificity. Colonisation rates were very high (99.5% of 215 needles were colonised) compared to similar studies of pine needles, such as the study of Sieber et al. [74] in which the colonisation rates of *Pinus mugo* ssp. *uncinata* needles were < 40% or *P. sylvestris* in Scotland where they ranged from 33–100% depending on needle age [7]. Similar to the findings of Peršoh et al. [31] for *P. sylvestris*, Sordariomycetes (57%) was by far the dominant class, rather than Leotiomycetes (20%), which dominate many *Pinus* endophyte studies [7].

Compared to previous studies on endophytic fungal communities on Scots pine in Scotland, there was one particularly interesting observation. Prior to this and another study at one of the same sites [34], *Anthostomella* spp. had not been recorded occurring endophytically with pines in Scotland [7,16], although they are known to be associated with pines in needle litter or on cones [75,76,77]. The significant abundance of *Anthostomella* spp. isolated in the present study (accounting for 52% of total isolates) was surprising, given the limited records on *Pinus* in the UK. *Anthostomella pinea* and the closely related *A.* aff. *pinea* largely dominated the fungal communities at each site and were isolated from each of the tree species. Little overlap in sites occurred between these taxa, which were distinct in colony colour (pink, *A. pinea*; white, *A.* aff. *pinea*) and manner of growth (Figure 5), and in sequence data (Figure 4). The culture characteristics described for the type of *A. pinea* [78] agree with those of *Anthostomella* aff. *pinea* (morphotype 22, Tentsmuir): white with a moderate aerial mycelium, a slightly woolly surface, and feathery margins [78]. However, phylogenetically, *A. pinea* collected in northeast France appears closer to *A. pinea* (morphotype 6 with pink colonies, Torrs Warren). In addition, the type of *A. pinea* is morphologically and phylogenetically similar to collections on dead Corsican pine litter needles at Torrs Warren (AM1, E01278543; Figure 4 and Figure 5), but single spore isolations of AM1 differ in culture characteristics, with it resembling *A. pinea* (morphotype 6). The morphological characteristics for AM1 include fully immersed, inconspicuous ascomata on paler areas of the dead needle, raising the host surface slightly, darker immediately surrounding the ostiole (*c.* 450 μm from above, 290 × 300 μm (h × w) in section), clypeus reduced to the ostiolar area; peridium 12–20 μm wide, wider near the ostiole and comprising dark compressed cells outwardly, becoming hyaline inwardly; the hymenium arises from the base of the ascoma and comprises hyphal paraphyses (2–2.5 μm), septate, embedded in a gel matrix; asci 74–150 × 9–11 μm, cylindrical, 8-spored, J-, short stipitate, becoming swollen and appearing compartmentalised; ascospores ellipsoidal, inequilateral, 16–18 × (6–)7–7.5 μm, bi-celled with a colourless, basal dwarf cell (2–3 μm long by 2.5 μm wide) and a larger brown cell (up to 15 μm long) with a 3/4 length germ slit and surrounded by a wide mucilaginous sheath (7–8.5 μm).

*Anthostomella* sp. has 100% ITS sequence matches with several pine-associated species including *A. formosa* (MW240652) and *Pseudoanthostomella pini-nigrae* (MW240654) (Figure 4; Supplementary Materials Table S2) and has over 99.8% ITS sequence identity/query coverage with several strains named *A. conorum* in GenBank (KT149745, MK188934, EU552099). However, their identity is unclear as no *A. conorum* strains have been previously isolated from spores and some of the aforementioned strains were not from pine hosts. This taxon was only found at Tentsmuir and accounted for 18% of isolates at the site. It was recovered from all three tree species, with more isolated from lodgepole pine. Cultures were distinctly circular and white with a very fine, felty appearance, which agreed with the description of *A. formosa* by Minter [77]. However, this contrasts with culture descriptions for the species by Lu & Hyde [79]: light brown, felty mycelia with a dentate edge, dark brown reverse, and staining agar brown. *Anthostomella conorum sensu* Lu & Hyde [79] matches the cultural characteristics of isolates in this study.

Virtually all 26 UK collection records of *Anthostomella* with *Pinus* in the British Mycological Society Fungal Record Database of Great Britain and Ireland (FRDBI) are derived from two studies of *Anthostomella* on needle litter conducted by Francis [76] and Minter [80], with only a further 7 records in CATE: https://cate.fungustrust.org.uk/records/index.php (accessed 25 September 2024). Francis [76] describes the genus on mainland Scotland, and the study by Minter [80] is a study of the mycobiota of the Scottish Isles of Rhum and Canna. Francis [76] points out that these fungi are rarely collected, and few exsiccati are available in herbaria. This situation is unlikely to have changed in the last 40 years. *Anthostomella* is not uncommon on Pinaceae (e.g., [77]), and it can have a widespread distribution where these hosts occur, with records from Europe, Asia and North America; however, it is rather under-recorded probably due to its extremely inconspicuous, immersed fruit bodies [77] (Figure 5). In Europe, *Anthostomella* species have been recorded as occurring endophytically on plantation pines such as in Serbia [30] and Spain [81] and also Poland where they occur in needles of native (*P. sylvestris*; often dominating the endophyte community) [11,82] as well as non-native pines (*P. nigra*; also one of the most abundant endophytic species) [21,83]. In Polish studies, there is a large overlap in the species diversity of dominant species of endophytic fungi with the present study (*Anthostomella* spp., *Cenangium ferruginosum*, *Desmazierella acicola* Lib. (=*Verticicladium trifidum* Preuss), *Lophodermium* spp., *Sydowia polyspora* (=*Sclerophoma pithyophila* (Corda) Höhn.). Kowalski [11] pointed out that despite the high levels of *Anthostomella* occurring endophytically, it is uncommon as a saprobe, occurring in one study [84] in less than 0.1% or 0.5% of litter and dead-attached Scots pine needles, respectively. This corresponds with the observations in the UK and is perplexing given the dominance of this species in its endophytic stage. It is worth pointing out that comparison in the present study of *Anthostomella* species with previous research is difficult, with no genetic data available for the latter. For instance, a surprising finding was the presence (and dominance) of *Anthostomella pinea*, not previously recorded in Britain; a species described in France in 2010 [78]. *Anthostomella pinea* shows morphological similarities with *A. formosa* Kirschst (see Figure 5) [75,76,77], but molecular data suggest that they are different (Figure 4), although the available sequences are not from the type of *A. formosa.* Unfortunately, the taxonomy of *Anthostomella* is acknowledged to be confused [56] and requires efforts to properly circumscribe the genus and clarify the species.

### 4.2. The Effects of Site, Tree Species, and Needle Age

Previous studies have shown that distinctive endophyte communities are associated with the same hosts at different sites (e.g., [16,18,85,86,87,88]). This was similarly demonstrated in the present study (Table 2, Figure 3; Supplementary Materials Figure S9) with differential colonisation of the same tree species at different sites. The ANOVA supported the significance of site for the total number of isolates per needle and the mean number of taxa isolated, the latter of which was dependant on the combined effect of site and tree species (Figure 1A,B; Supplementary Materials Tables S4 and S5). In assessing isolate distribution, all of the main taxa, with the exception of *L. seditiosum,* were found in much greater abundance at one or the other site and had varying degrees of host tree species preference (Table 2). This showed that endophyte composition varied depending on site but that the unique site–tree species interactions were not fixed and did not produce equitable effects. The CA ordination (Figure 3) illustrates this, showing the clear separation of Torrs Warren and Tentsmuir at opposing poles in dimension one. Within these site distinctions, the unique assemblages of endophytes correspond to each tree species, whereas dimension two distinguishes Scots pine (native, subsection *Pinus*) and lodgepole pine (non-native, subsection *Contortae*), with Corsican pine (non-native, subsection *Pinus*) overlapping this axis. These results might reflect the relatedness of the pine species and their geographical distribution and origin, shown through greater similarity of endophytic fungal communities in more phylogenetically related tree hosts. Subsection *Pinus* is almost exclusively found in Eurasia, with Scots pine and Corsican pine having a slightly overlapping distribution, whereas subsection *Contortae* occurs in North America and Mexico [89]. The Sørensen–Dice Index of Similarity values for tree species across combined sites support this observation, indicating that Scots pine–Corsican pine were most similar in terms of fungal endophyte diversity. A greater similarity of endophyte communities has previously been recorded between Scots and Corsican pine, in comparison with another European *Pinus*, *P. pinaster*, but which occurs in a different subsection (*Pinaster*) [90]. This echoes the findings of Hata & Funai [20] who found that endophyte communities were influenced by host taxonomic affinities. However, although the fungal endophyte community recorded in the study presented herein closely resembled those in studies on Scots pine and Austrian pine (*P. nigra*) by Kowalski and colleagues [21,82,83], there is little known about the foliar endophytic fungi of lodgepole pine, with only metabarcoding studies available for comparison [91].

Factors influencing endophytic fungal abundance, diversity and community composition are not well understood. Local habitats provide hosts with distinct pools of available endophytic fungi and potential fungal inoculum [22]. These habitats are also affected by a spectrum of complex factors which influence endophyte composition, such as land use history, vegetation composition, degree of adaptation to local mycoflora, and various abiotic factors [20,22]. A further complicating factor when trying to disentangle host and site influences in the present study is that the sites are all plantations. Prior to planting, both of the plantations in this study existed as unforested dune areas or grazing lands. As plantations, Tentsmuir and Torrs Warren each have a distinct microhabitat which may harbour considerably different endophyte communities compared to natural tree populations. The overall dominance of *Anthostomella* spp. (Sordariomycetes), as opposed to *Lophodermium* spp. (Leotiomycetes), may reflect this. Notably, studies of endophytic communities of Scots pine in Caledonian pine forests [7] or common gardens [16] remote from non-native pines have consistently lacked *Anthostomella* spp. and suggest that there has potentially been naturalisation of possible European fungal endophyte species (e.g., *Anthostomella* spp.) in line with the findings in mixed, native/non-native plantations in Poland [11,21,82,83] and elsewhere [30].

Torrs Warren and Tentsmuir had the most obvious contrast of significantly different precipitation levels, a known influence on endophyte abundance and dispersal, which along with temperature, influence germination and growth of fungal species but affect them differentially depending on the species’ biology. Martínez-Álvarez et al. [90] noted that climatic variables corresponded with the endophyte colonisation patterns of *Pinus* hosts, namely winter precipitation and mean minimum temperature. Although Torrs Warren (the wetter site) did not have higher colonisation rates, as shown in some previous conifer studies [18], there were notable differences in community composition between sites.

Particular variance between the site and tree species interactions (as illustrated in Figure 1A,B) may be explained by a range of factors. The highest mean taxa per needle sample was found in Corsican pine at Torrs Warren. This may be due to the fact that Corsican pine made up 90% of the original plantation composition in the 1950s. A correspondingly large reservoir of endophytic fungal inoculum and associated diversity may have accumulated, particularly in Corsican pine needle litter. Lodgepole pine and Scots pine were later additions to the site and planted in smaller numbers, a fact which may have contributed to the dominance of a single taxon, and lower diversity in each case. The abundance of *A. pinea* in lodgepole pine (77%) resulted in high mean isolates per sample (9.00), but low H’ (0.917). Scots pine in TW was confined to a very small population, also dominated by one taxon, *L. seditiosum* (and with an unexpected lack of *L. pinastri*), and potentially compromised as indicated by the lack of year-two needles. In Tentsmuir, the much larger site (1569 hectares), Scots pine and Corsican pine were planted in the 1920s, with the later addition of lodgepole pine. The latter species made up by far the smallest component at the site and likewise had the lowest mean endophyte taxa (1.92) and mean isolates (4.03) per needle sample. This was in contrast with more taxa and isolates recovered from Tentsmuir for Scots pine and Corsican pine. This situation points to the diminished availability of potential endophytic fungal colonisers for lodgepole pine. It also may suggest that the species is less adapted than its counterparts to the microhabitat and associated influences present at Tentsmuir.

Tree species, as noted to a degree above, was significant for mean taxa per needle in ANOVA, although an overall dominance of site specificity over host specificity was detected in this study. Interestingly, it has been suggested that the occurrence of a taxon on multiple host species does not necessarily signify equality in terms of interaction with those hosts [92]. It is also possible that species specificity is not necessarily inherent in fungal endophyte taxa, but rather that it is sometimes a function of environment [22]. Host species presents a unique combination of factors including subtle differences in chemical composition, PH and even genotype [16,93], which undoubtedly contributed to the formation of the distinct endophyte assemblages found in this study.

Endophytic fungi occur in greater abundance on older leaves or needles due to longer exposure to potential inoculum and physical changes of plant tissue over time including cuticle degradation, which allows for easier penetration [7,9,18,94,95]. While needle age did not have a significant influence, distribution patterns emerged for individual taxa with *Lophodermium pinastri* only inhabiting year-two needles and high rates of colonisation of older needles by *Clypeosphaeria* sp. and Xylariaceae sp. (29/31). However, the dominant taxa, *Anthostomella* spp., had the opposite tendency and inhabited younger needles with greater frequency (68% occurring in year-one needles). This agrees with previous observations [11,21] and may indicate that *Anthostomella* spp. have poor competitive abilities. It could also point to a difference in the nutrient availability of older needles [11].

## 5. Conclusions

While some endophyte community differences in this study reflected the native (SP) vs. non-native (CP and LP) status of host tree species, it did not explain the complex overall pattern observed. The results were more aligned with previous work showing that taxonomically proximal host trees have more similar endophyte communities than those that are more distantly related [18,20]. While this theory does not totally fit the results of this study, it provides a more meaningful explanation for the patterns. Hata & Futai [20] in their study of 45 species and sub-species of *Pinus* showed a clear similarity of endophyte colonisation according to the taxonomic group of the host, conducted at a single site. The observations made in the present study show that the site can have a powerful and changeable interaction with a given host. The similarity detected in the study by Hata & Futai [20] could certainly have changed with the site, as the level of adaptation and many other factors are implicated in endophyte recruitment.

An unexpected finding in the present study was the dominance of *Anthostomella* spp. and the great difficulties encountered in taxonomic assignment. Some taxonomic revision of *Anthostomella* in the UK seems warranted but unfortunately could not be undertaken in this study due to the lack of specimens as mainly only endophytes were sampled. In addition, the DNA of the *Anthostomella* endophyte isolates was not available, so further sequencing was not possible. The only publications available on the genus are heterogeneous and disagree on the ascospore characteristics of *A. formosa* (which the *A. pinea* on needle litter was originally presumed to be) [76,77,79]. It is likely that there are many species of *Anthostomella* on *Pinus* in the UK. No records for *A. pinea* could be found in the UK to date, and published records of it occurring endophytically are available only for *Fraxinus excelsior* [96] or as an endolichen [97]. This is possibly not surprising though, given its recent publication, the inconspicuous nature of this taxon, and the lack of clarity regarding the taxonomy.

## Figures and Tables

**Table 1:** Fungal strains used in the phylogenetic analysis in this study. *Tub*2 sequences were not included due to the quality of some of the sequence data available. * = type specimens. Newly generated sequences from the present study are shown in bold.

Species	Strain	Host	Location	ITS	LSU
*Anthostomella formosa*	MFLUCC_14_0170	*Pinus sylvestris*	Italy	MW240652	KP340544
Anthostomella obesa *	MFLUCC_14_0171	*Cornus* sp.	Italy	KP297405	KP340546
Anthostomella pinea *	CBS_128205	*Pinus* sp.	France	HQ599578	-
*Anthostomella pinea*	AM1.1	*Pinus nigra* subsp. *Laricio*	Scotland (Torrs Warren)	**PQ895305**	**PQ900125**
*Anthostomella pinea*	6_1	*Pinus contorta*	Scotland (Torrs Warren)	**PQ895285**	-
*Anthostomella* aff. *pinea*	22_3	*Pinus sylvestris*	Scotland (Tentsmuir)	**PQ895282**	-
*Anthostomella* sp.	36_1	*Pinus sylvestris*	Scotland (Tentsmuir)	**PQ895288**	-
*Antostomella rubicola*	MFLUCC_16_0479	*Cornus sanguidea*	Italy	KX533455	KX533456
Microdochium lycopodinum *	CBS 122885	*Lycopodium annotinum*	Austria	JF440979	JF440979
Microdochium phragmitis *	CBS 285.71	*Phragmites australis*	Poland	KP859013	KP858949
*Pseudoanthostomella delitescens*	MFLUCC_16-0477	*Pinus nigra*	Italy	KX533451	KX533452
Pseudoanthostomella pini-nigrae *	MFLUCC_16_0478	*Pinus nigra*	Italy	KX533453	KX533454
Pseudoanthostomella senecionicola *	MFLUCC_15_0013	*Senecio* sp.	Italy	MW240674	KX505959
Endophyte	ARIZ_B328	*Pinus ponderosa*	Arizona, USA	MZ015842	-

**Table 2:** Distribution of endophytic fungal taxa at each site for each pine species (with totals). SP = Scots pine; CP = Corsican pine; LP = lodgepole pine. * = infrequent morphotypes or overgrown/contaminated isolates; morphotype number codes appear in brackets.

Taxonomic Assignment	Torrs Warren			Total	Tentsmuir			Total
	SP	CP	LP		SP	CP	LP	
*Anthostomella pinea* (6)	45	94	263	**402**	0	6	0	**6**
*Anthostomella* aff. *pinea* (22)	5	4	8	**17**	21	72	39	**132**
*Anthostomella* sp. (36)	0	0	0	**0**	25	32	55	**112**
*Cenangium ferruginosum* (16)	0	2	0	**2**	0	18	0	**18**
*Clypeosphaeria* sp. (26)	0	0	6	**6**	1	24	6	**31**
*Desmazierella acicola* (3, 11)	6	48	26	**80**	3	7	1	**11**
*Dothistroma septosporum* (2)	0	0	0	**0**	0	0	2	**2**
*Lophodermium pinastri* (15)	0	0	0	**0**	0	0	14	**14**
*Lophodermium seditiosum* (4, 19, 27, 25, 39, 33)	38	12	1	**51**	103	61	7	**171**
*Preussia*-like (9)	0	1	3	**4**	0	3	0	**3**
*Sydowia polyspora* (7, 21)	0	1	1	**2**	3	4	2	**9**
*Xylaria* sp. (8, 14, 18)	3	13	8	**24**	5	4	1	**10**
Xylariaceae sp. (29, 31)	1	3	8	**12**	29	5	1	**35**
Indeterminate * (32, 10, 1)	10	12	11	**33**	10	25	5	**40**
Unidentified sp. 12	0	0	0	**0**	3	3	16	**22**
Unidentified sp. 17	0	3	0	**3**	1	4	0	**5**
Unidentified sp. 20	1	0	1	**2**	0	0	1	**1**
Unidentified sp. 23	0	0	0	**0**	1	3	0	**4**
Unidentified sp. 24	0	0	0	**0**	3	4	1	**8**
Unidentified sp. 28	0	1	0	**1**	1	0	2	**3**
Unidentified sp. 30	0	0	2	**2**	4	0	0	**4**
Unidentified yeast (35)	0	1	0	**1**	1	3	1	**5**
Totals	109	195	338	**642**	214	278	154	**646**

## Data Availability

The sequence data presented in this study are openly available in [GenBank].

## Supplementary Materials

The following supporting information can be downloaded at: https://www.mdpi.com/article/10.3390/jof11020148/s1 Figure S1. Location of sample sites; Figure S2. Torrs Warren Wood; Figure S3. GPS locations Torrs Warren; Figure S4. Pine plantation at Torrs Warren; Figure S5. Tentsmuir Forest; Figure S6. GPS locations Tentsmuir; Figure S7. Pine tree Tentsmuir; Figure S8. Contributions of row and column values for Correspondence Analysis factor map; Figure S9. The distribution of four dominant endophyte taxa by tree and site; Table S1. GPS Coordinates for trees sampled; Table S2. ITS sequence data; Table S3. Number of isolates and distribution of taxa across sites; Table S4. Descriptive statistics for mean number of isolates per needle; Table S5. Descriptive statistics for mean fungal taxa per needle; Table S6. Shannon diversity index (H′) results; Table S7. Sørensen-Dice index of similarity results.
