# Supplementary material for: Foliar Fungal Endophyte Communities of Scottish Plantation Pines
Source: J Fungi (Basel). 2025 Feb 14;11(2):148. doi: 10.3390/jof11020148 (PMC11856089; doi:10.3390/jof11020148)
Supplement: Supplementary file 1 [file jof-11-00148-s001.zip › jof-3392461-supplementary.pdf]

Supplementary Material Figures

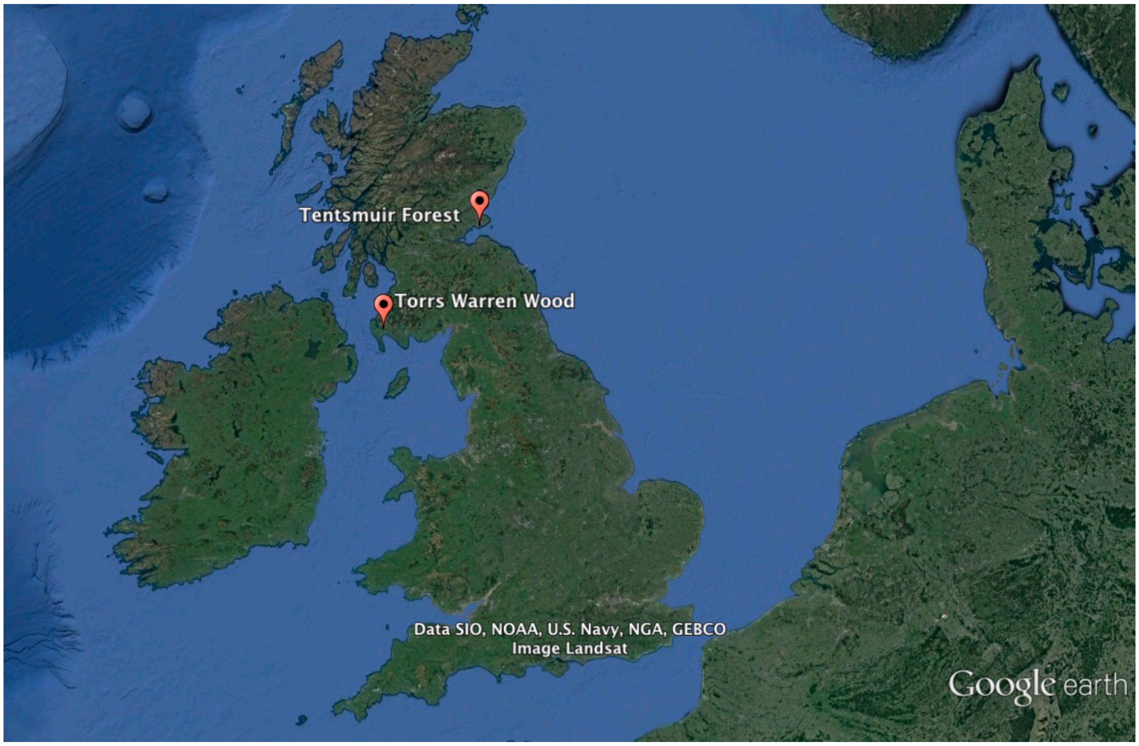

Supplementary Material Figure S1. Location of sample sites on opposing coasts in Scotland.

Torrs Warren Forest

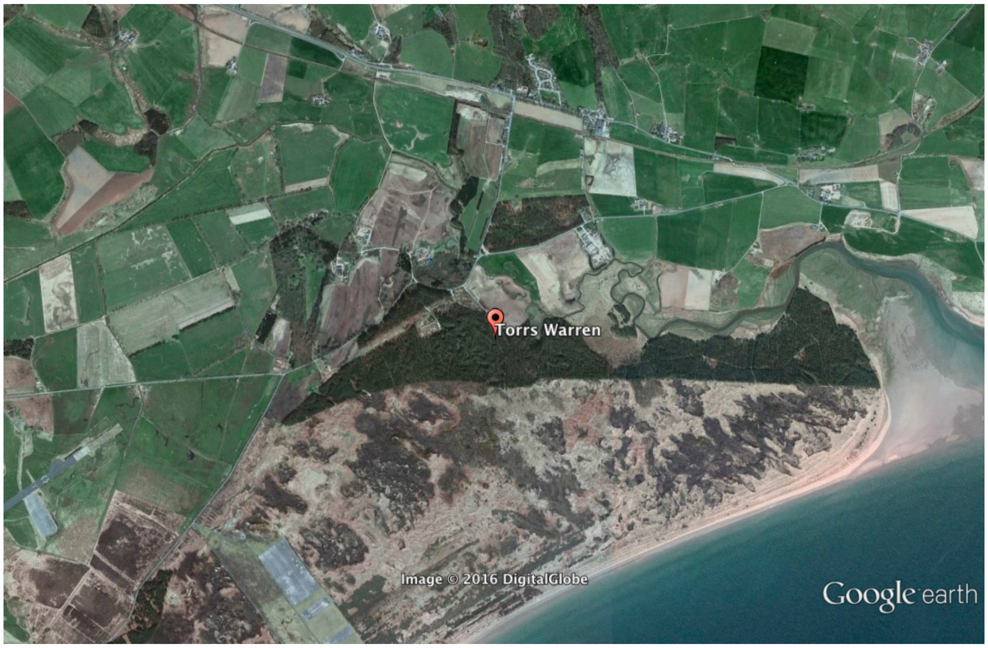

Supplementary Material Figure S2. Torrs Warren Wood with high dune area in foreground

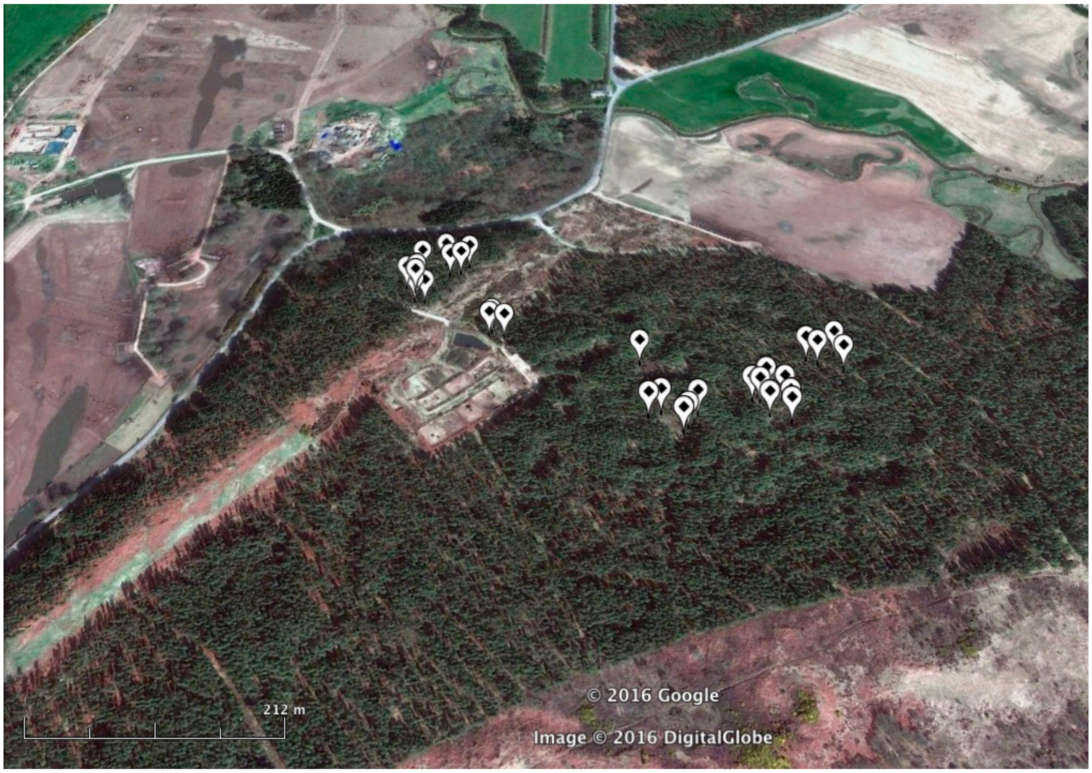

Supplementary Material Figure S3. GPS locations marked for 30 trees sampled in Torrs Warren Wood

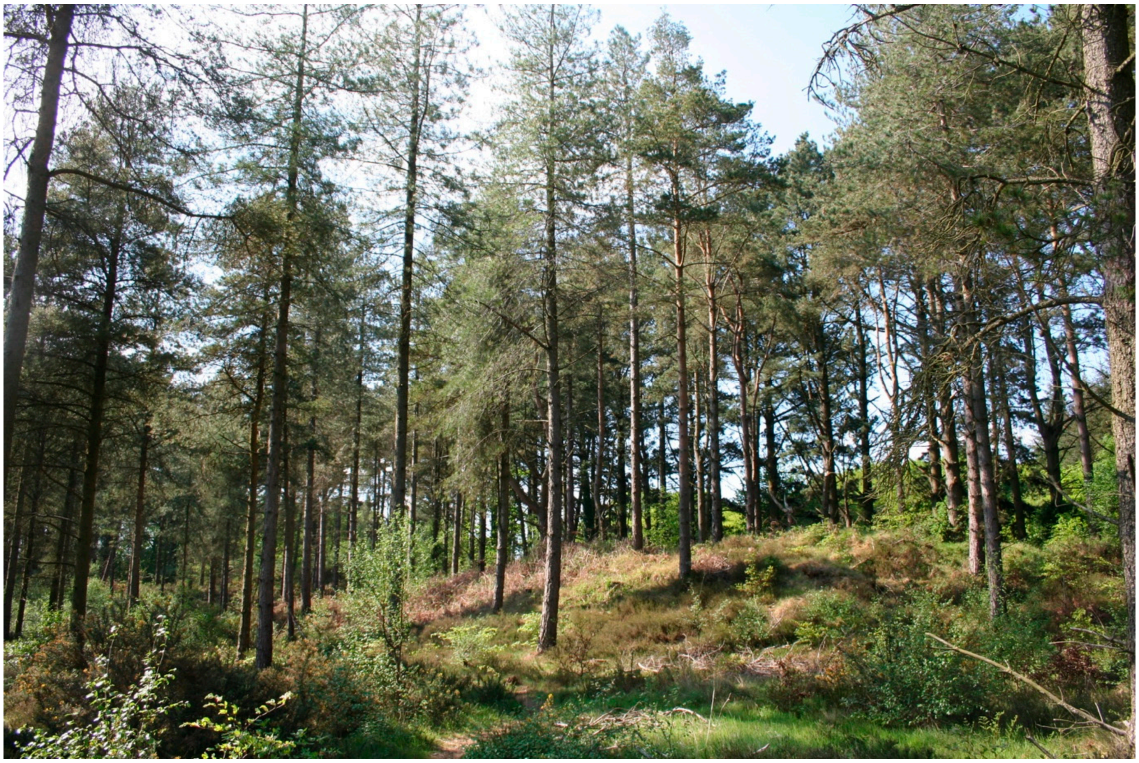

Supplementary Material Figure S4. The open hilly aspect of mixed pine plantation with dense ground cover in Torrs Warren Wood

Tentsmuir Forest

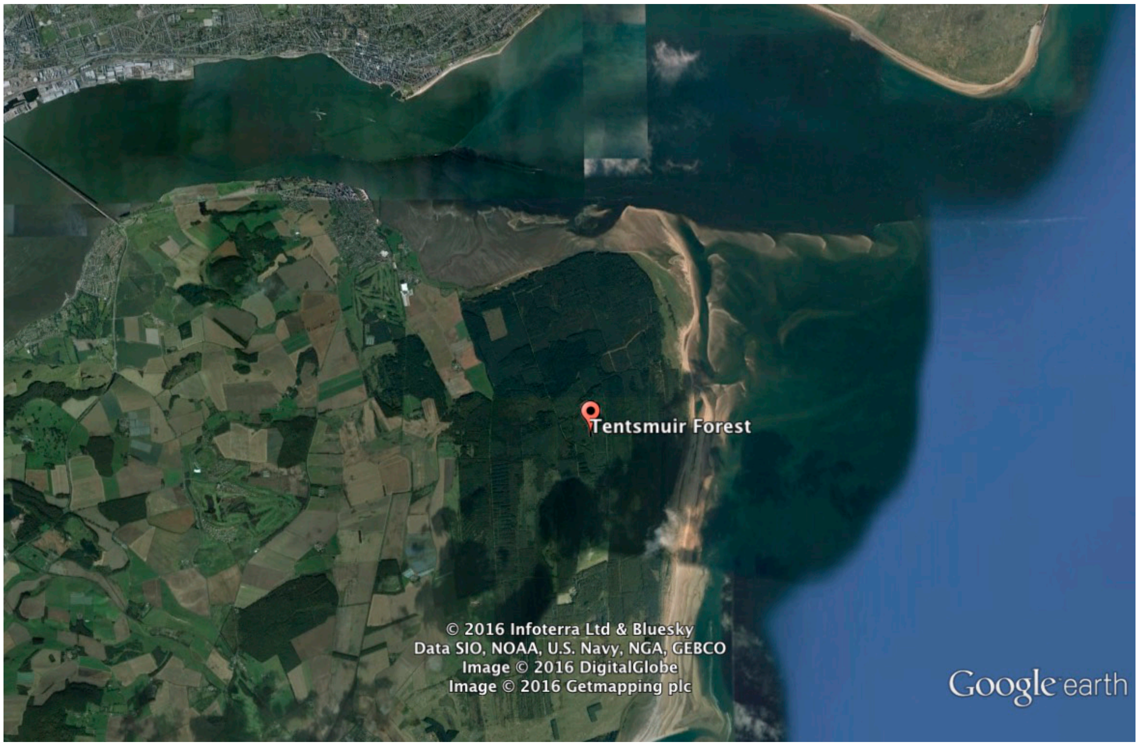

Supplementary Material Figure S5. Tentsmuir Forest and dune area

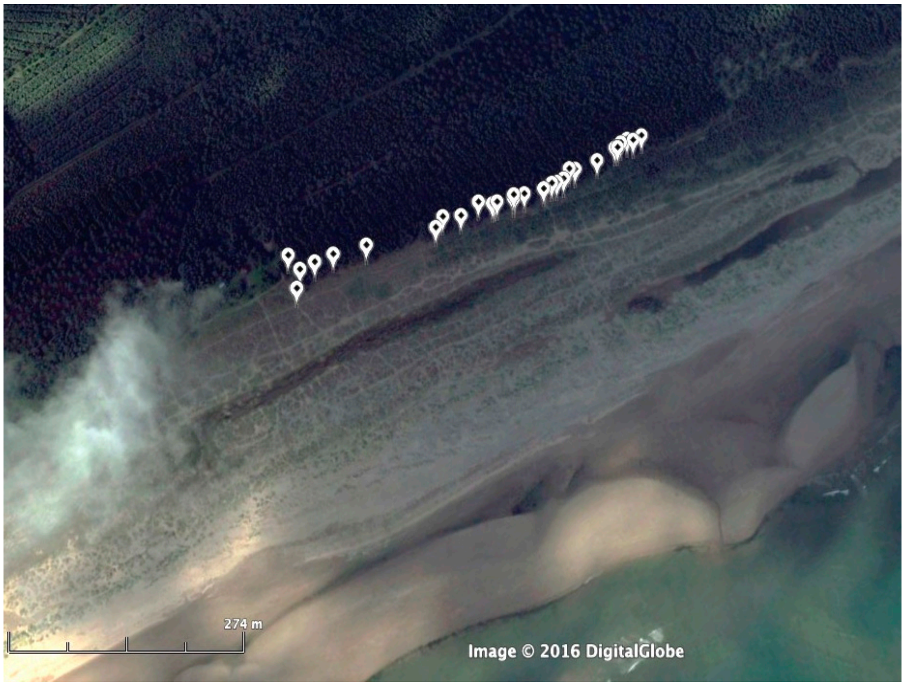

Supplementary Material Figure S6. GPS locations for 30 tree samples along Tentsmuir dunes

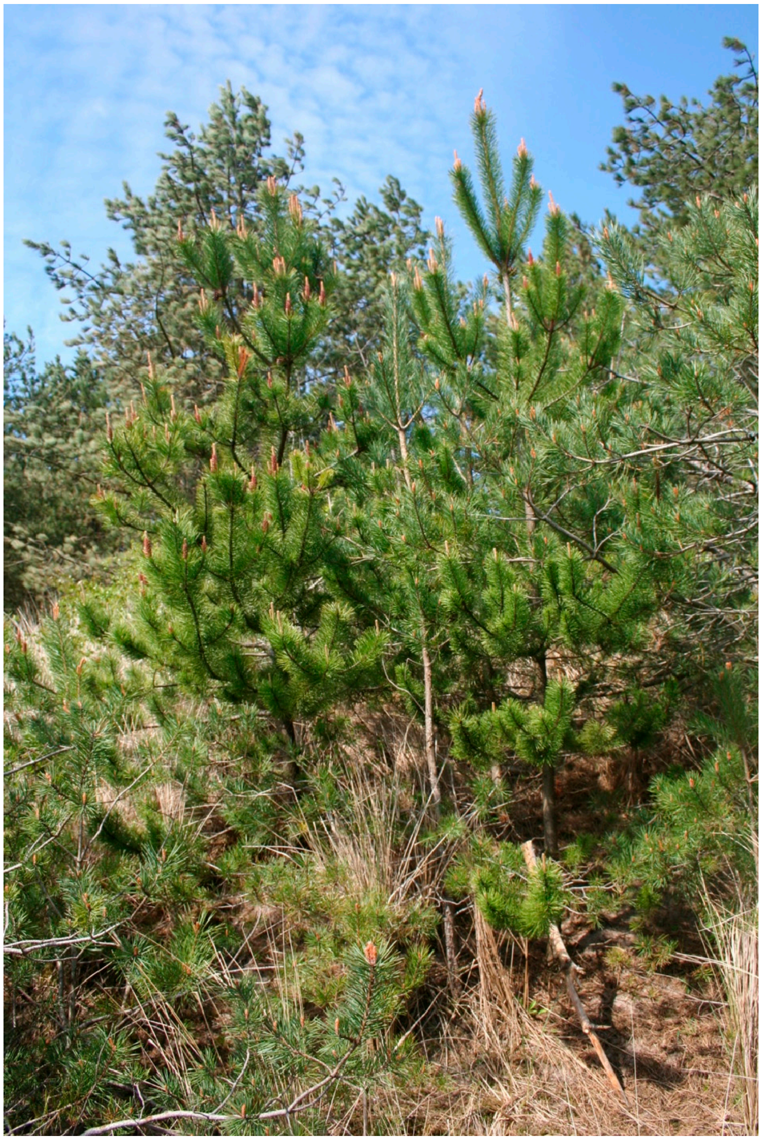

Supplementary Material Figure S7. The open, somewhat exposed mixed planting in Tentsmuir

Figure S8. Contributions of row (**Figure a**) and column (**Figure b**) values for Correspondence Analysis factor map

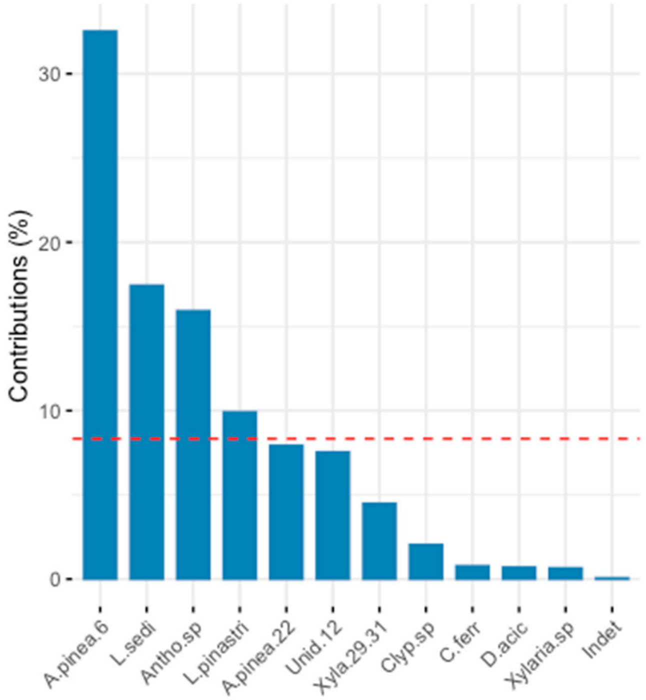

Figure a: Percent contribution of taxa (<1% colonization) rows to dimensions one and two in CA.

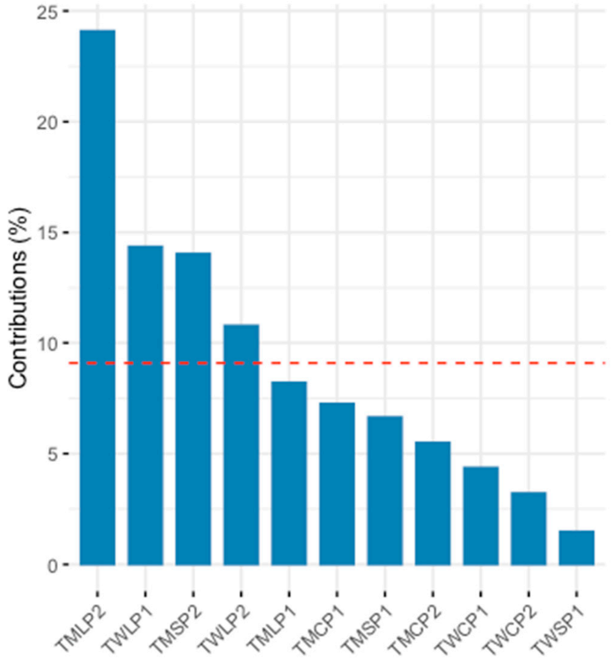

Figure b: Percent contribution of eleven site-species-needle age combinations to dimensions one and two in CA factor plot.

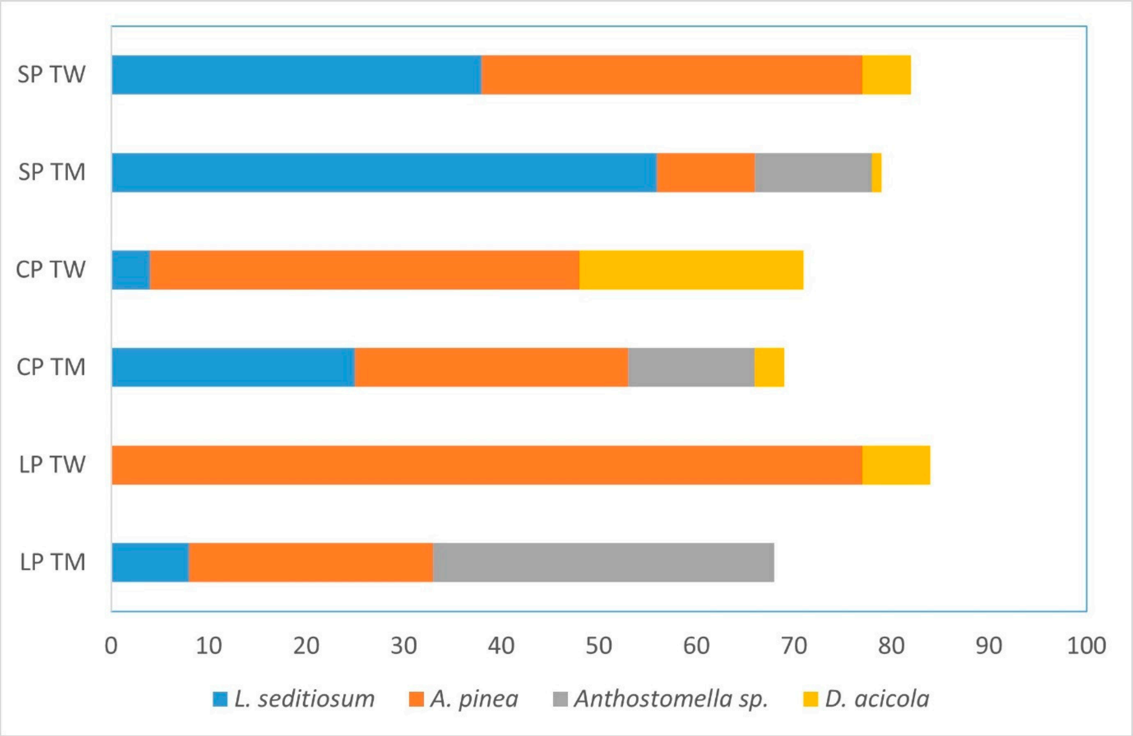

Supplementary Material Figure S9. The distribution of four dominant endophyte taxa calculated as the percentage of isolates per total of the given tree species at the given site. *Anthostomella pinea* and *A.* aff. *pinea* are represented as a single taxon, with site specificity implied for each site. LP shows the biggest contrast in dominant taxa. TM = Tentsmuir; TW = Torrs Warren; CP = Corsican pine; LP = lodgepole pine; SP = Scots pine.
