# Supplementary material for: Foliar Fungal Endophyte Communities of Scottish Plantation Pines
Source: J Fungi (Basel). 2025 Feb 14;11(2):148. doi: 10.3390/jof11020148 (PMC11856089; doi:10.3390/jof11020148)
Supplement: Supplementary file 1 [file jof-11-00148-s001.zip › Supplementary Material Tables.pdf]

Supplementary Material Table S1. GPS Coordinates for trees sampled in Torrs Warren (TW) and Tentsmuir (TM) Forests. CP = Corsican pine; LP = lodgepole pine; SP = Scots pine.

Torrs Warren	*°Lat. (N)*	*°Long. (W)*	*Tentsmuir*	*°Lat. (N)*	*°Long. (W)*
TW-SP1	54.86677	-4.89550	TM-SP1	56.41378	-2.81062
TW-SP2	54.86672	-4.89568	TM-SP2	56.41537	-2.80872
TW-SP3	54.86673	-4.89587	TM-SP3	56.41570	-2.80862
TW-SP4	54.86689	-4.89588	TM-SP4	56.41608	-2.80844
TW-SP5	54.86668	-4.89650	TM-SP5	56.41633	-2.80827
TW-SP6	54.86686	-4.89632	TM-SP6	56.41669	-2.80805
TW-SP7	54.89667	-4.89667	TM-SP7	56.41687	-2.80800
TW-SP8	54.86660	-4.89655	TM-SP8	56.41771	-2.80787
TW-SP9	54.86647	-4.89661	TM-SP9	56.41780	-2.80788
TW-SP10	54.86642	-4.89645	TM-SP10	56.41807	-2.80775
TW-CP1	54.86418	-4.89027	TM-CP1	56.41377	-2.81008
TW-CP2	54.86417	-4.89167	TM-CP2	56.41398	-2.80964
TW-CP3	54.86406	-4.89186	TM-CP3	56.41421	-2.80954
TW-CP4	54.86389	-4.89182	TM-CP4	56.41458	-2.80927
TW-CP5	54.86376	-4.89153	TM-CP5	56.41551	-2.80883
TW-CP6	54.86398	-4.89323	TM-CP6	56.41595	-2.80864
TW-CP7	54.86424	-4.89344	TM-CP7	56.41647	-2.80821
TW-CP8	54.86567	-4.89536	TM-CP8	56.41696	-2.80797
TW-CP9	54.86575	-4.89559	TM-CP9	56.41715	-2.80799
TW-CP10	54.86578	-4.89553	TM-CP10	56.41790	-2.80787
TW-LP1	54.86432	-4.89064	TM-LP1	56.31800	-2.80966
TW-LP2	54.86441	-4.89077	TM-LP2	56.41365	-2.80936
TW-LP3	54.86437	-4.89028	TM-LP3	56.41611	-2.80843
TW-LP4	54.86411	-4.89197	TM-LP4	56.41614	-2.80840
TW-LP5	54.86385	-4.89149	TM-LP5	56.41636	-2.80833
TW-LP6	54.86402	-4.89146	TM-LP6	56.41682	-2.80805
TW-LP7	54.86412	-4.89289	TM-LP7	56.41709	-2.80807
TW-LP8	54.86402	-4.89313	TM-LP8	56.41743	-2.80786
TW-LP9	54.86424	-4.89365	TM-LP9	56.41774	-2.80783
TW-LP10	54.86489	-4.89341	TM-LP10	56.41795	-2.80776

Supplementary Material Table S2. ITS sequence comparison query results with best matches from two databases; '*' represents a unidirectional sequence and '**'denotes additional Genbank and UNITE 100% matches to: *Pseudoanthostomella senecionicola* (MW240674); *Pseucoanthostomella pini-nigrae* (MW240654); *Anthostomella formosa* (MW240652). '!' denotes incorrect morphotyping. Table ordered by EDNA number.

Collection no. (morphotype no.-replicate no.)	EDNA number	UNITE result	%	Accession	GenBank result	%	Accession no.	ITS sequence Accession no. (present study)
TW-LP5A-1 (22-1)	EDNA16-0045214	*Anthostomella pinea*	96%	MF327371	*A. pinea*	96%	MF327371	PQ895273
TM-SP5A-2 (25-1)	EDNA16-0045215	*Lophodermium seditiosum*	100%	KY742565	*Lophodermium seditiosum*	100%	KY742565	PQ895274
TM-SP10-1 (25-2)	EDNA16-0045216	*L. seditiosum*	100%	UDB035451	*L. seditiosum*	100%	KY742559	PQ895275
TW-CP5-1 (16-1)	EDNA16-0045217	*Cenangium ferruginosum*	99%	UDB031061	*Cenangium ferruginosum*	99%	KY742574	PQ895276
TM-SP6-1 (25-3)	EDNA16-0045218	*L. seditiosum*	100%	UDB035451	*L. seditiosum*	100%	KY742559	PQ895277
TM-SP8-1 (19-1)	EDNA16-0045219	*L. seditiosum*	100%	UDB035451	*L. seditiosum*	100%	KY742559	PQ895278
TM-SP5A-1 (27-1)	EDNA16-0045220	*L. seditiosum*	100%	UDB035451	*L. seditiosum*	100%	KY742559	PQ895279
TW-SP4A-1 (4-1)	EDNA16-0045221	*L. seditiosum*	100%	UDB035451	*L. seditiosum*	100%	KY742559	PQ895280
TW-LP3-2 (22-2)*	EDNA16-0045235	*A. pinea*	94%	OK576226	*A. pinea*	95%	NR190906	PQ895281
TM-SP1-1 (22-3)	EDNA16-0045236	*A. pinea*	96%	MF327371	*A. pinea*	96%	MF327371	PQ895282
TW-SP1A-1 (22-4)	EDNA16-0045237	*A. pinea*	100%	OK576226	*A. pinea*	100%	NR190906	PQ895283
TW-CP2-1 (22-5)!	EDNA16-0045238	*Atractidochium hillariae*	100%	KM519290	*Atractidochium hillariae*	100%	NR158306	PQ895284
TW-LP4A-1 (6-1)	EDNA16-0045239	*A. pinea*	100%	OK576226	*A. pinea*	100%	NR190906	PQ895285

TW-SP9A-1 (6-2)	EDNA16-0045240	*A. pinea*	100%	OK576226	*A. pinea*	100%	NR190906	PQ895286
TW-CP3A-1 (6-3)	EDNA16-0045241	*A. pinea*	99%	OK576226	*A. pinea*	100%	NR190906	PQ895287
TM-SP8-1 (36-1)	EDNA16-0045242	*'A. conorum' (Psuedoanthostomella conorum)*	100%	KT149745**	*'A. conorum' (Psuedoanthostomella conorum)*	100%	KT149745**	PQ895288
TM-SP3-1 (36-2)	EDNA16-0045243	*'A. conorum' (Psuedoanthostomella conorum)*	100%	KT149745**	*'A. conorum' (Psuedoanthostomella conorum)*	100%	KT149745**	PQ895289
TM-CP3A-2 (36-3)	EDNA16-0045244	*'A. conorum' (Psuedoanthostomella conorum)*	100%	KT149745**	*'A. conorum' (Psuedoanthostomella conorum)*	100%	KT149745**	PQ895290
TM-SP4A-1 (26-1)!	EDNA16-0045245	*A. pinea*	96%	MF327371	*A. pinea*	96%	MF327371	PQ895291
TW-LP5-1 (26-2)	EDNA16-0045246	*Clypeosphaeria sp.*	97%	KT949898	*Clypeosphaeria mamillana*	97%	KT949898	PQ895292
TM-CP9A-1 (26-3)	EDNA16-0045247	*Clypeosphaeria sp.*	97%	KT949898	*C. mamillana*	97%	KT949898	PQ895293
TM-LP6-1 (26-4)	EDNA16-0045248	*Clypeosphaeria sp.*	97%	KT949898	*C. mamillana*	97%	KT949898	PQ895294
TM-CP4-2 (11-1)	EDNA16-0045249	*Desmazierella acicola*	100%	OP699790	*Desmazierella acicola*	100%	OP699789	PQ895295
TW-LP8A-1 (11-2)	EDNA16-0045250	*D. acicola*	100%	OP699790	*D. acicola*	100%	OP699789	PQ895296
TW-SP6A-1 (3-1)	EDNA16-0045251	*D. acicola*	100%	OP699790	*D. acicola*	100%	OP699789	PQ895297
TW-SP10A-1 (4-2)	EDNA16-0045252	*L. seditiosum*	100%	UDB035451	*L. seditiosum*	100%	KY742559	PQ895298
TW-CP9A-2 (4-3)	EDNA16-0045253	*L. seditiosum*	100%	UDB035451	*L. seditiosum*	100%	KY742559	PQ895299
TM-SP6A-1 (4-4)	EDNA16-0045254	*L. seditiosum*	100%	UDB035451	*L. seditiosum*	100%	KY742565	PQ895300
TM-CP1-2 (4-5)	EDNA16-0045255	*L. seditiosum*	100%	KY742565	*L. seditiosum*	100%	KY742565	PQ895301

TW-CP4-2 (16-2)	EDNA16-0045256	*C. ferruginosum*	99%	PP523927	*C. ferruginosum*	99%	PP523927	PQ895302
TM-CP2A-1 (25-4)	EDNA16-0045257	*L. seditiosum*	100%	KY742565	*L. seditiosum*	100%	KY742565	PQ895303
TW-SP3-1 (27-2)	EDNA16-0045258	*L. seditiosum*	100%	KY742565	*L. seditiosum*	100%	KY742565	PQ895304
TWs-AM1.1	EDNA16-0046604	*A. pinea*	100%	OK576226	*A. pinea*	100%	NR190906	PQ895305
TWs-AM1.2	EDNA16-0046605	*A. pinea*	100%	OK576226	*A. pinea*	100%	NR190906	PQ895306
TWs-AM1.3	EDNA16-0046606	*A. pinea*	100%	OK576226	*A. pinea*	100%	NR190906	PQ895307
TWs-AM1.4	EDNA16-0046607	*A. pinea*	100%	OK576226	*A. pinea*	100%	NR190906	PQ895308

Supplementary Material Table S3. Number of isolates and distribution of taxa across sites. * = note that second year needles were absent on Scots pine at Torrs Warren, and were therefore not collected. TW = Torrs Warren; TM = Tentsmuir; SP = Scots pine; LP = lodgepole pine; CP = Corsican pine.

Site	Tree sp.	Total isolates	No. of taxa
TW	SP*	109	8
	CP	195	13
	LP	338	12
	Total:	**642**	**16**
TM	SP	214	16
	CP	278	17
	LP	154	16
	Total:	**646**	**22**
Both sites	SP	323	16
	CP	473	19
	LP	492	21
	Total:	**1288**	**22**

Supplementary Material Table S4. Descriptive statistics for mean number of isolates per needle for each tree species at each site (above); N = number of needle samples; CP = Corsican pine; LP = lodgepole pine; SP = Scots pine. ANOVA GLM (below) using the same parameters as in Table S4, with total isolates as response. (showing: degrees of freedom, adjusted sum of squares, adjusted mean of squares, F-statistic, and probability value).

Site	***Sp.***	***N***	***Mean***	***SE Mean***	***SD***
Torrs Warren					
	SP	20	5.700	0.603	2.697
	CP	39	5.462	0.470	2.937
	LP	38	9.000	0.822	5.067
Tentsmuir					
	SP	40	5.325	0.311	1.966
	CP	38	6.737	0.498	3.073
	LP	39	4.026	0.457	2.851

Source	***DF***	***Adj. SS***	***Adj. MS***	***F-Value***	***P-value***
Site	1	90.20	90.200	8.14	0.006
Species	2	28.17	14.084	1.27	0.288
Needle age	1	0.08	0.083	0.01	0.930
Site*species	2	383.15	191.573	17.28	0.000
Tree (site,species)	54	600.76	11.125	1.05	0.403
Error	153	1624.25	10.616	----	----

Supplementary Material Table S5. Descriptive statistics for mean fungal taxa per needle for each tree species at each site (above); N = number of needle samples; CP = Corsican pine; LP = lodgepole pine; SP = Scots pine. ANOVA GLM (below)Response= ‘taxa’, fixed factors: ‘species’, ‘site’, ‘needle age’; random factor: ‘tree’ (showing: degrees of freedom, adjusted sum of squares, adjusted mean of squares, F-statistic, and probability value).

Site	***Sp.***	***N***	***Mean***	***SE Mean***	***SD***
Torrs Warren					
	SP	20	2.250	0.204	0.910
	CP	39	2.385	0.186	1.161
	LP	38	2.368	0.143	0.883
Tentsmuir					
	SP	40	2.475	0.139	0.887
	CP	38	3.237	0.162	0.998
	LP	39	1.923	0.124	0.774

Source	***DF***	***Adj. SS***	***Adj. MS***	***F-Value***	***P-value***
Site	1	1.847	1.84729	1.75	0.191
Species	2	16.954	8.47682	8.00	0.001
Needle age	1	0.014	0.01442	0.02	0.895
Site*species	2	16.818	8.40911	7.95	0.001
Tree (site,species)	54	58.179	1.07739	1.30	0.113
Error	49	127.236	0.83161	----	----

Supplementary Material Table S6. Shannon diversity index (H'), values were calculated for a single population for each tree species at each site, and then each tree species across combined sites. CP = Corsican pine; LP = lodgepole pine; SP = Scots pine.

Site	Species	H′
Torrs Warren	SP	1.490
	CP	1.595
	LP	0.917
Tentsmuir	SP	1.660
	CP	2.096
	LP	1.856
Both sites	SP	1.880
	CP	2.249
	LP	1.784

Supplementary Material Table S7. Sørensen-Dice index of similarity, values were calculated using the number of fungal taxa per tree to compare the fungal communities of two tree species in terms of shared and unique endophytic fungi for each site and across combined sites. CP = Corsican pine; LP = lodgepole pine; SP = Scots pine.

Site	Species	S-D
Torrs Warren	SP—CP	0.782
	SP—LP	0.761
	CP—LP	0.833
Tentsmuir	SP—CP	0.774
	SP—LP	0.774
	CP—LP	0.688
Both sites	SP—CP	0.914
	SP—LP	0.894
	CP—LP	0.857
